# Ultrasonically Modified Amended-Cloud Point Extraction for Simultaneous Pre-Concentration of Neonicotinoid Insecticide Residues

**DOI:** 10.3390/molecules23051165

**Published:** 2018-05-12

**Authors:** Rawikan Kachangoon, Jitlada Vichapong, Rodjana Burakham, Yanawath Santaladchaiyakit, Supalax Srijaranai

**Affiliations:** 1 Creative Chemistry and Innovation Research Unit, Department of Chemistry and Center of Excellence for Innovation in Chemistry, Faculty of Science, Mahasarakham University, Maha Sarakham 44150, Thailand; wittisit@gmail.com; 2 Materials Chemistry Research Center, Department of Chemistry and Center of Excellence for Innovation in Chemistry, Faculty of Science, Khon Kaen University, Khon Kaen 40002, Thailand; rodjbu@kku.ac.th (R.B.); supalax@kku.ac.th (S.S.); 3 Department of Chemistry, Faculty of Engineering, Rajamangala University of Technology Isan, Khon Kaen Campus, Khon Kaen 40000, Thailand; yanawath.sa@rmuti.ac.th

**Keywords:** dual-cloud point extraction, high-performance liquid chromatography, neonicotinoid insecticides, water samples

## Abstract

An effective pre-concentration method, namely amended-cloud point extraction (CPE), has been developed for the extraction and pre-concentration of neonicotinoid insecticide residues. The studied analytes including clothianidin, imidacloprid, acetamiprid, thiamethoxam and thiacloprid were chosen as a model compound. The amended-CPE procedure included two cloud point processes. Triton™ X-114 was used to extract neonicotinoid residues into the surfactant-rich phase and then the analytes were transferred into an alkaline solution with the help of ultrasound energy. The extracts were then analyzed by high-performance liquid chromatography (HPLC) coupled with a monolithic column. Several factors influencing the extraction efficiency were studied such as kind and concentration of surfactant, type and content of salts, kind and concentration of back extraction agent, and incubation temperature and time. Enrichment factors (EFs) were found in the range of 20–333 folds. The limits of detection of the studied neonicotinoids were in the range of 0.0003–0.002 µg mL^−1^ which are below the maximum residue limits (MRLs) established by the European Union (EU). Good repeatability was obtained with relative standard deviations lower than 1.92% and 4.54% for retention time (*t_R_*) and peak area, respectively. The developed extraction method was successfully applied for the analysis of water samples. No detectable residues of neonicotinoids in the studied samples were found.

## 1. Introduction

Neonicotinoids have been commercialized as a new generation of pesticides affecting the central nervous system of insects, whilst they exert a lower neurotoxicity towards mammals than previously developed insecticides [1]. Neonicotinoids were developed as a safer and more effective class of pesticides [2], and as a result they have become the most popular group of insecticides with applications in agriculture and veterinary medicine [1] instead of other classes of pesticides, including organochlorines, organophosphates and carbamates. This group of insecticides includes nitro-substituted (dinotefuran, nitenpyram, thiamethoxam, imidacloprid and clothianidin) and cyano-substituted (acetamiprid and thiacloprid) compounds [3]. They are intended for the treatment of a wide range of plants including sunflower, corn, canola, cotton, potato, rice, sugar beets, oil rapeseed, soy, ornamental plants, tree nurseries and fruits [4]. Neonicotinoid insecticides act as agonists at the insect nicotinic acetylcholine receptors (nAChRs), which play an important role in synaptic transmission in the central nervous system [5]. The European Commission has banned the use of imidacloprid, thiamethoxam and clothianidin in crops attractive to pollinators in the next two years emphasizing awareness of the potential harmful impact of the neonicotinoids on honeybees and their products [3]. Therefore, a highly sensitive and selective analytical method for monitoring neonicotinoid residues at low concentration levels is required to secure food quality and to protect the consumer from hazards.

Neonicotinoid insecticides are unsuitable for direct analysis by gas chromatography due to their low volatility and high polarity [6]. Capillary electrophoresis (CE) has also become an attractive approach for the separation of insecticide residues, however the main disadvantage of CE is its poor concentration sensitivity due to the short optical length of the capillary [7]. As a result, high-performance liquid chromatography (HPLC) coupled with various detection systems, including electrochemical (ECD) [8], fluorescence (FLD) [9], ultraviolet (UV)/diode array (DAD) [3,10] and mass spectrometry (MS) [11], is the favored technique for multi-analysis of neonicotinoid pesticides. The C18 column was generally used for separation of some neonicotinoids in various matrices. HPLC has been developed on fast separation through the use of sub-2 µm particle columns, fused-core particle columns, or monolithic columns [12]. Monolithic columns show some potential advantages that are very attractive for medical and biological fields [13]. These columns are characterised by a different structure than the conventional packed ones. The monolithic packing consists of a single continuous piece of either an organic polymer or silica, with a bimodal porous structure comprising macropores and mesopores [14,15]. The macropores or throughpores (typically 2 µm) are responsible for a higher permeability allowing the use of high flow rates at considerably reduced backpressure, while the mesopores (about 13 nm) provide a large surface area which makes possible a good interaction with analytes [12]. Therefore, faster separations can be achieved without sacrificing chromatographic efficiency [14].

The absence of clean-up steps require a suitable chromatographic separation in order to avoid the coelution of potential interferents, and pre-concentration of the target analytes. Despite this, the limits of detection (LODs) of these methods are still low enough to detect neonicotinoid insecticides at the concentration levels of actual regulatory limits. Cloud point extraction (CPE) is an alternative and powerful method for pre-concentration and separation of pesticides [16,17], comprising green chemistry [18]. CPE is based primarily on the phase behavior of non-ionic surfactants in aqueous solutions, which exhibit phase separation after an increase of temperature more than the cloud point temperature of each surfactant. The drawbacks of traditional CPE are overcome by the dual-cloud point extraction (d-CPE) method [19]. The removal of interfering species through the d-CPE procedure improves the efficiency and selectivity of the proposed method, and the back extraction of analyte in aqueous solution is naturally compatible with the conditions of instrumental analysis. This technique includes two cloud point extraction processes; the first part of d-CPE is done as traditional CPE, and finally the analyte is back-extracted into the alkaline aqueous phase in the second cloud point step [20]. In this case, d-CPE was employed to eliminate the effect of surfactant on injection and separation [21]. A d-CPE was developed for the analysis of salfonamide [22], auxins [21], mercury [19], copper [23] and uranium [24].

The aim of this study is to establish an ultrasonically modified amended-cloud point extraction following the analysis by HPLC with a monolithic column for the simultaneous pre-concentration of neonicotinoid insecticide residues. Triton^TM^ X-114 (Bellefonte, USA) was used to extract neonicotinoid insecticides into the surfactant-rich phase and then the analytes were back extracted with alkaline solution to overcome the surfactant unpropitious effects and to increase the selectivity and efficiency of the proposed method. The parameters affecting the amended-CPE (such as kind and concentration of surfactant, type and content of salts, kind and concentration of back extraction agent, and incubation temperature and time) for the extraction and pre-concentration of target analytes as well as HPLC with monolithic column conditions were also investigated.

## 2. Results and Discussion

### 2.1. Optimization of Ultrasonically Modified Amended-Cloud Point Extraction Procedure

Several factors which affect the ultrasonically modified amended-cloud point extraction procedure such as kind and concentration of surfactant, salt addition, kind and concentration of back extraction agent, and incubation temperature and time, were optimized to obtain the maximum extraction efficiency of the proposed method. In this study, these factors were evaluated one factor at a time while the other remaining factors were kept constant. The optimization was carried out on the aqueous solution (10 mL) containing 0.50 µg mL^−1^ of each analyte. All the experiments were performed in triplicate and the mean of the results were used for optimization.

#### 2.1.1. Effect of Salt Addition

**Figure 1:**
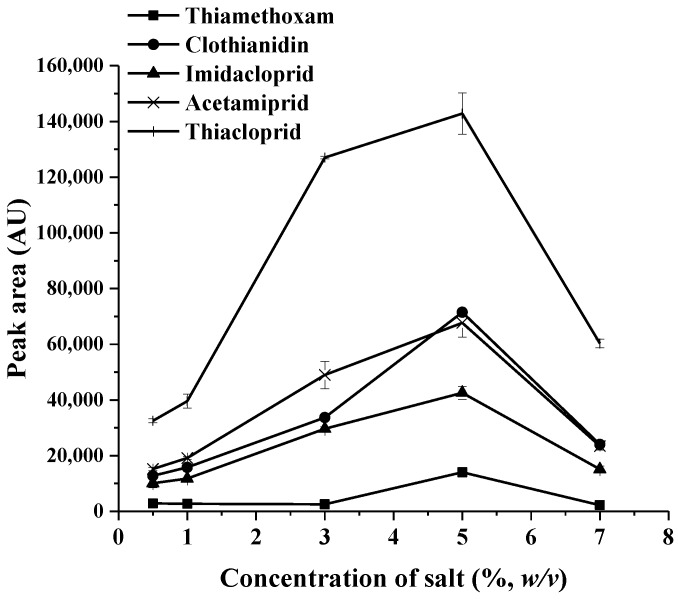
Effect of amount of salt on the extraction recovery (0.50 µg mL^−1^) of each neonicotinoid). AU: Absorbance unit.

In general, the addition of salt could decrease the solubility of aqueous sample phase and lead to enhancement of their partitioning of the analytes into the surfactant rich phase (SRP) by the “salting out” phenomenon. The presence of salt can increase the incompatibility between the water structures in the hydration shells of analytes and surfactant macromolecules, which can reduce the concentration of “free water” in the surfactant-rich phase and, consequently, reduce the volume of the phase [17]. Different electrolyte salts (i.e., NaCl, Na_2_SO_4_, Na_2_CO_3_, and CH_3_COONa) at the amount of 3% (*w/v*) were investigated and compared with no salt addition. It was found that Na_2_SO_4_ gave higher peak area for most analytes; Na_2_SO_4_ was then studied in the amount of 0.5–7% (*w/v*). As shown in Figure 1, the results demonstrate that the highest response in terms of peak area was obtained when the amount of Na_2_SO_4_ was 5% (*w/v*). After that, the peak area decreased. A high salt amount increases aqueous viscosity, thus opposing the migration of the target analytes into the extraction phase. Therefore, 5% (*w/v*) of Na_2_SO_4_ was selected for further experiments.

#### 2.1.2. Effect of Concentration of Triton^TM^ X-114

**Figure 2:**
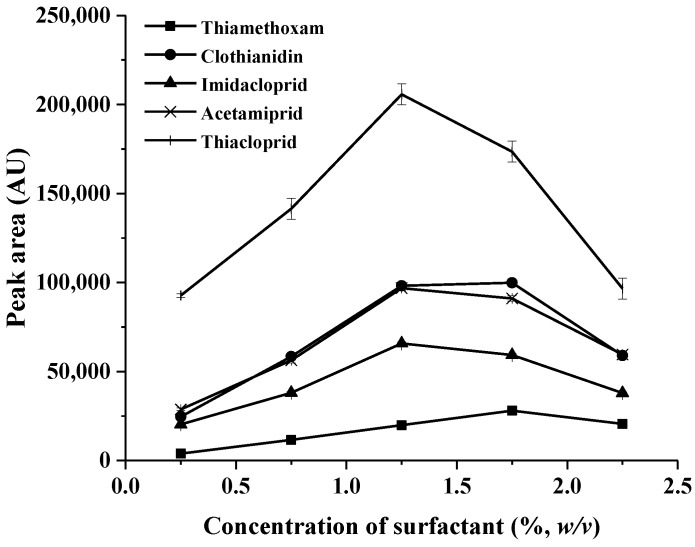
Effect of concentration of surfactant on the extraction recovery (0.50 µg mL^−1^) of each neonicotinoid).

Triton^TM^ X-114 was selected as surfactant in a subsequent experiment due to its low cloud point temperature (≈ 22 to 23 °C) and low critical micelle concentration (≈ 0.2 mM) [25] and can easily settle down in the aqueous phase after centrifugation, due to a high density that facilitates phase separation. In this study, the concentration of Triton^TM^ X-114 in aqueous solution was evaluated in the range of 0.25–2.25% (*w/v*). As shown in Figure 2, the highest peak area was observed when the concentration of Triton^TM^ X-114 was 1.25% (*w/v*). This is due to the increase in the volume of surfactant-rich phase (SRP) that dilute the target analytes in the final volumes. For further study, the concentration of Triton^TM^ X-114 at 1.25% (*w/v*) was selected as optimum.

#### 2.1.3. Effect of Kind and Concentration of Back Extraction Agent

**Figure 3:**
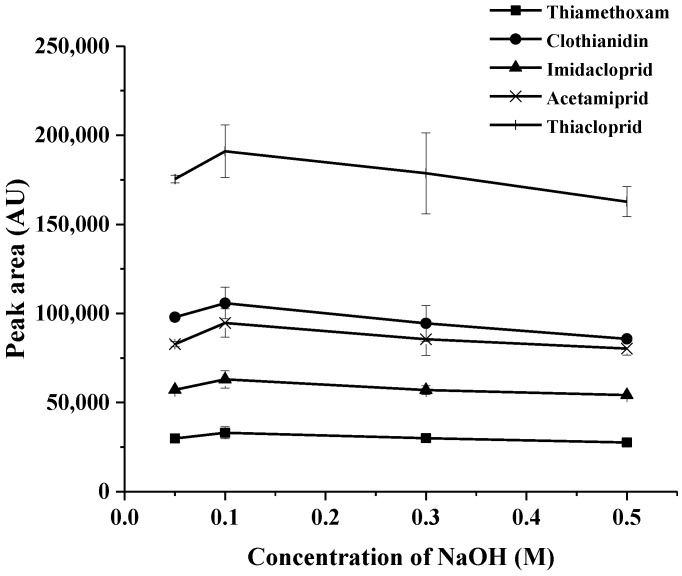
Effect of concentration of NaOH on the extraction recovery (0.50 µg mL^−1^) of each neonicotinoid).

In the second phase of CPE, two types of back extraction agent were studied. For this propose, basic (NaOH) and acidic (HCl) reagents of 0.05–1.0 M were evaluated for the back extraction of the study analytes in the aqueous phase of their hydrophobic complexes being trapped in micellar media. It was found that NaOH exhibits an excellent extraction efficiency of back extraction agent (data not shown). Therefore, NaOH was chosen as the back extraction agent. As shown in Figure 3, the extraction efficiency is the highest when the concentration of NaOH is 0.1 M. Consequently, NaOH 0.1 M was selected for further study.

#### 2.1.4. Effect of Incubation Temperature and Time

Theoretically, the optimal incubation temperature of the CPE occurs when the equilibration temperature is higher than the cloud point temperature of the surfactant [17,26,27]. Triton^TM^ X-114 was used in subsequent experiments because it has low cloud-point temperature (≈ 22 to 23 °C) [25]. In the present study, the effect of incubation temperature was carried out in an ultrasonic bath at the temperature range of 25–60 °C. As the operating temperature increases, the volume of the coacervate phase decreases because of the dehydration occurs in the surfactant rich phase [28]. This can be interpreted to mean that as the temperature increases, the hydrogen bonds are disrupted and dehydration occurs [29]. The highest extraction efficiency in term of peak area was observed at 30 °C (data not shown). The effect of incubation time on the extraction efficiency was studied in the range of 1 to 30 min. It was found that, the maximum efficiency was obtained at 3 min of incubation time (data not shown). It is desirable to use the shortest incubation time and the lowest possible incubation temperature as a compromise between the completion of extraction and efficient separation of the phases [30]. Therefore, 30 °C of incubation temperature and 3 min of incubation time were selected for further study.

### 2.2. Analytical Performance of the Proposed Method

**Figure 4:**
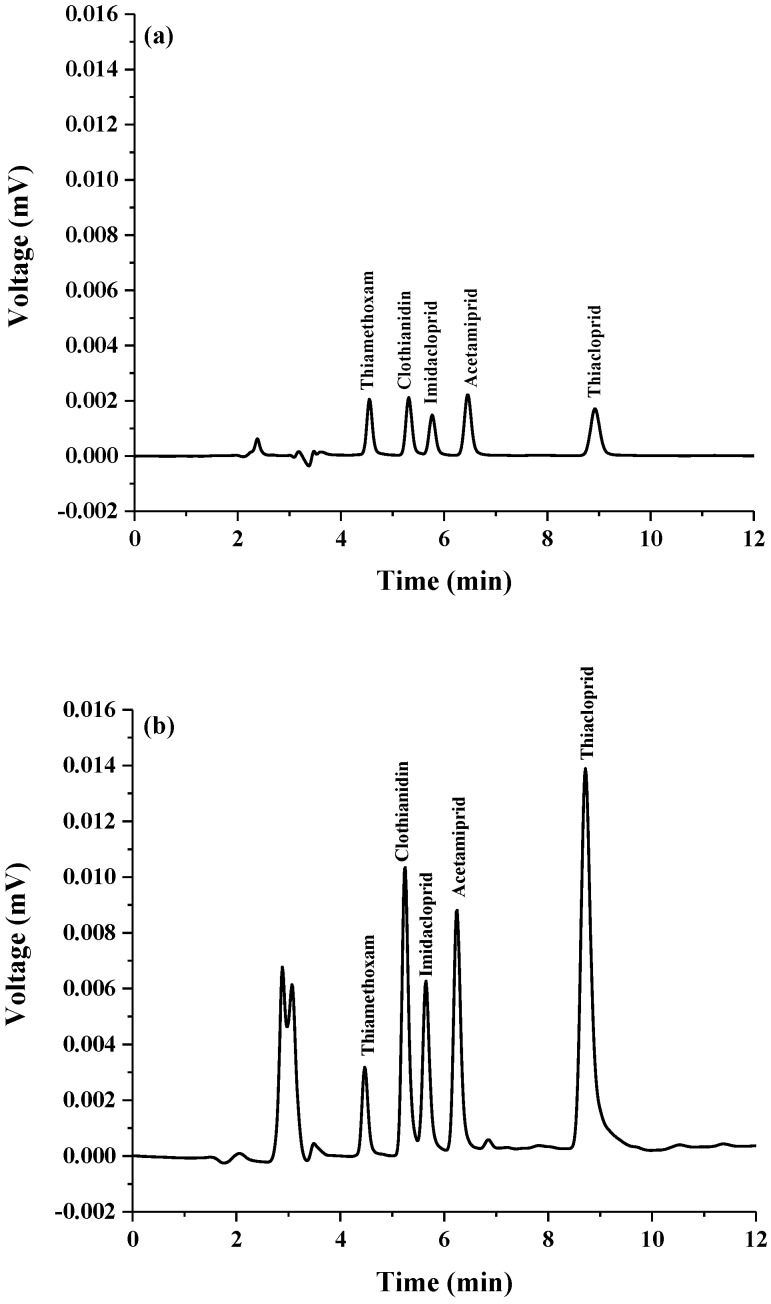
Chromatograms of standard neonicotinoids obtained by (**a**) without pre-concentration: concentration of all standards was 0.50 µg mL^−1^, (**b**) with the ultrasonically modified amended-CPE method: concentration of all standards was 0.50 µg mL^−1^.

After optimization of the developed method, the analytical performance was evaluated in terms of linearity, precision, limit of detection (LOD), limit of quantification (LOQ) and enrichment factor. The experimental results are summarized in Table 1. Calibration curves were constructed by triplicate injections of standard solution of neonicotinoid insecticides at eight concentrations obtained after extraction. The peak area of each neonicotinoid was plotted against its concentration. LOD and LOQ were defined as the concentration of the target analytes giving signal-to-noise ratio (S/N) = 3 and 10, respectively. The precision in terms of intraday (*n* = 5) and over several days (*n* = 5 × 3 days) was also investigated as the relative standard deviation (RSD) of retention time (*t_R_*) and peak area of the studied compounds. Good linearities covered the range of 0.005–0.7 µg mL^−1^ with a correlation coefficient (*r*^2^) ≥ 0.9978. LOD were in the range of 0.0003–0.002 µg mL^−1^. The enrichment factor (EF), defined as the ratio of slope of calibration obtained from the proposed extraction method to that without pre-concentration, was also investigated. The obtained EFs were 20–333-fold. High precision was observed with RSD lower than 2.00 (*t_R_*) and 5.00 (peak area), respectively. These results show that the developed extraction method coupled to HPLC can increase the sensitivity of detection (see Figure 4) and provide high precision.

### 2.3. Application to Real Samples

**Figure 5:**
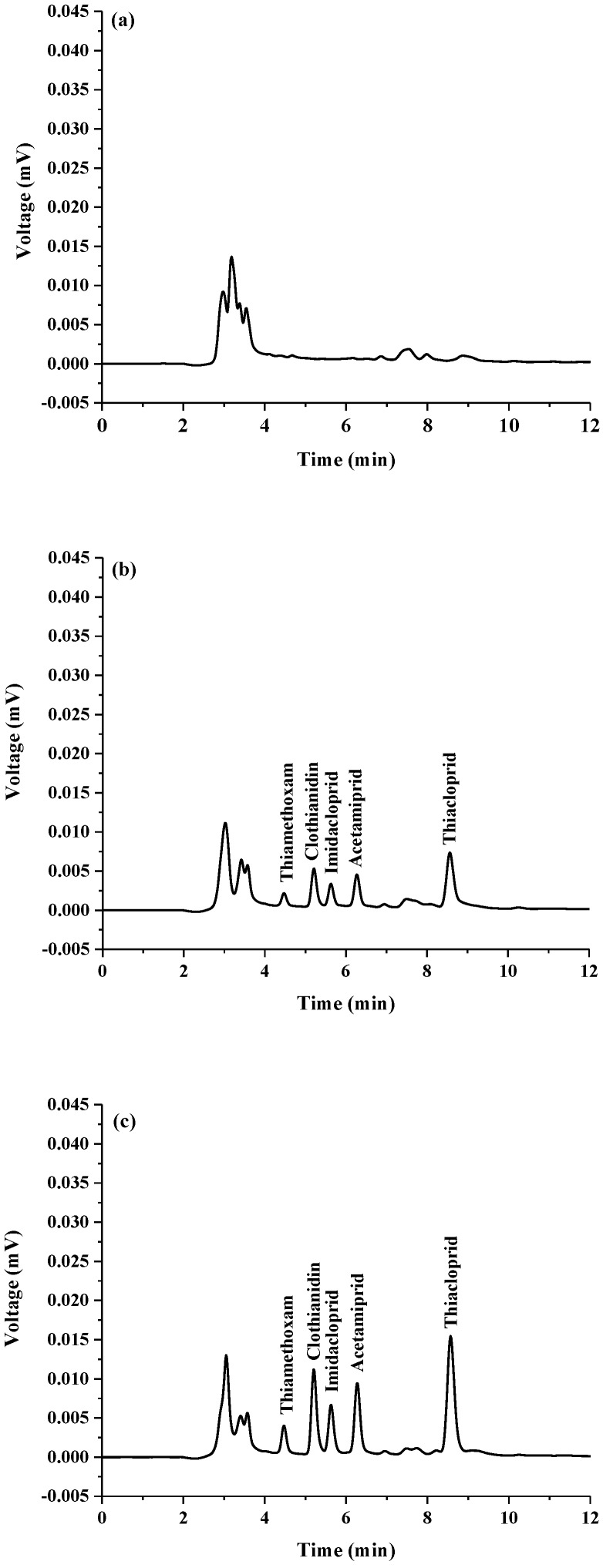

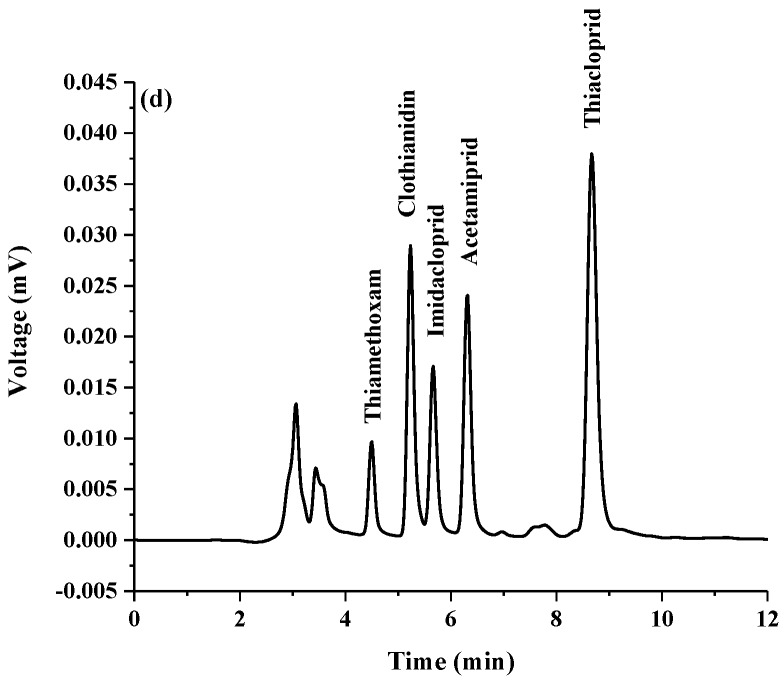
The chromatograms of (**a**) surface water sample, (**b**) spiked surface water sample at 0.05 µg mL^−1^, (**c**) spiked surface water sample at 0.10 µg mL^−1^ and (**d**) spiked surface water sample at 0.25 µg mL^−1^.

The proposed method was applied for the determination of neonicotinoid residues in surface water samples. The result is shown in Table 2. It was found that no neonicotinoid insecticide residues were observed in any surface water samples studied. The accuracy of the proposed method was evaluated in term of % recovery. The surface water samples were spiked with the target insecticides at different concentrations of 0.05, 0.10 and 0.25 µg mL^−1^, before extraction and analysis. Extractions were carried out under the optimum extraction conditions and recoveries are summarized in Table 2. Accuracy (percentage values) from matrix-matched calibration were in the range of 60.11–119.63% with their respective RSD lower than 13.71%. The chromatograms of blank and spiked surface water samples at three concentration levels are shown in Figure 5.

### 2.4. Comparison with Other Sample Preparation Methods

A comparison of the present ultrasonically modified amended-cloud point extraction method with other sensitive methods such as dispersive liquid-liquid microextraction (DLLME), dispersive solid-phase extraction (DSPE) and room-temperature ionic liquid-liquid-phase microextraction (RTIL-LPME) for the determination of some neonicotinoid insecticide residues in water samples is provided in Table 3. It was found that the proposed method provides high sensitivity in term of low LODs and high EFs. However, one of the main drawbacks of quick, easy, cheap, effective, rugged and safe (QuEChERS) methodology is the fact that there is no pesticide concentration step in the final extract while toxic organic solvent is required to extract the target analytes [31]. The proposed method has been recognized as an alternative to the conventional extraction because of its performance, lower cost and lower toxicity because a surfactant is used as an extractant. Another significant advantage of the proposed method is that it is a faster, simpler and a greener method with a requirement of a small volume of sample.

## 3. Materials and Methods

### 3.1. Chemicals and Reagents

All reagents were analytical grade or higher. Five standards of neonicotinoid insecticide including clothianidin, imidacloprid, acetamiprid, and thiamethoxam were obtained from Dr. Ehren-storfer GmbH (Augsburg, Germany), and thiacloprid were purchased from Sigma-Aldrich (Taufkirchen, Germany). All standard neonicotinoid insecticides were of analytical standard grade. The stock solution of each insecticide (1000 µg mL^−1^) was prepared by dissolving an appropriate amount in methanol. Working standard solutions were prepared by diluting the stock standard solution with water. Chromatographic grade methanol and acetonitrile were purchased from Merck (Darmstadt, Germany). Sodium chloride (NaCl), anhydrous sodium sulphate (anh. Na_2_SO_4_), sodium carbonate (Na_2_CO_3_) and sodium hydroxide (NaOH) were obtained from Ajax Finechem (Auckland, New Zealand), Sodium acetate (CH_3_COONa) was obtained from Carlo Erba (Val de Reuil, France). Triton™ X-114 was obtained from Sigma-Aldrich (St. Louis, MI, USA). Sodium dodecyl sulfate (SDS) and Triton™ X-100 were provided by Merck. Cetyltrimethyl ammonium bromide (Calbiochem, Germany) was also used. Water used throughout the experiment was produced by RiOs^TM^ Type I Simplicity 185 (Millipore Waters, Burlington, MA, USA) with the resistivity of 18.2 MΩ.cm.

### 3.2. Instrumentation

For chromatographic separation, a Waters 1525 Binary LC system (Waters, Milford, MA, USA) equipped with a degasser, a column compartment, a manual injector, and a photo-diode array detector (PDA) was used. Empower 3 software was used for data acquisition. Separation was performed on a Chromolith^®^ HighResolution RP-18 endcapped (4.6 × 100 mm) column (Merck). The injection volume was 20 µL and all of the studied insecticides were detected at 254 nm. The mobile phase consisted of acetonitrile and water (26:74, *v/v*) at a flow rate of 0.5 mL min^−1^. Five neonicotinoid insecticides were separated within 9 min with the elution order of thiamethoxam (*t_R_* = 4.54 min), clothianidin (*t_R_* = 5.30 min), imidacloprid (*t_R_* = 5.76 min), acetamiprid (*t_R_* = 6.45 min) and thiacloprid (*t_R_* = 8.91 min). A centrifuge (Centurion, UK) was used to complete phase separation. An ultrasonic bath (Dksh, Hamburg, Germany) and a vortex mixer (Fisher Scientific, Waltham, MA, USA) were also used.

### 3.3. Ultrasonically-Modified Amended-Cloud Point Extraction Procedure

**Figure 6:**
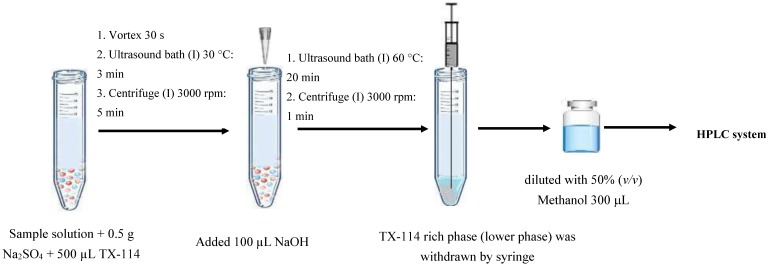
Schematic diagram of the proposed extraction method.

Figure 6 shows schematic diagram of the proposed extraction method prior to HPLC analysis. For the first CPE, 10 mL of real sample or standard solution was mixed with 5% (*w/v*) of Na_2_SO_4_ before subsequently transferring to a 15 mL screw cap test tube. After that, 1.25% (*w/v*) Triton™ X-114 was added into an aqueous solution before vortexing the tube for 30 s. Then the solution was immersed in an ultrasonic bath at 30 °C for 3 min. The phase separation was accelerated by centrifugation at 3000 rpm for 5 min. The target analytes in aqueous sample were extracted into the surfactant-rich phase settled at the bottom of the tube. The aqueous phase was removed carefully by using a syringe.

For the second CPE, 100 µL of 0.1 M NaOH was injected into the surfactant rich phase. The mixture was immersed in an ultrasonic bath at 60 °C for 20 min to form new two phases, and then the mixture was centrifuged at 3000 rpm for 1 min to separate the two resulting phases. The aqueous phase was diluted with 300 µL of 50% (*v/v*) methanol (the minimum amount necessary to completely dissolve the aqueous phase) to decrease viscosity and then passed through a 0.45 µm nylon membrane filter. Finally, 20 µL of the extract was directly injected into the HPLC.

## 4. Conclusions

This work demonstrates the application of the ultrasonically modified amended-cloud point extraction method for sample preparation and pre-concentration of some neoicotinoid insecticide residues prior to HPLC analysis. Triton™ X-114 was used to extract some neonicotinoid residues into the surfactant-rich phase and the target analytes were then transferred into an alkaline solution using an amended-CPE process. An ultrasound agitator was used for acceleration of the mass transfer in the extraction process. The proposed method provides high sensitivity, simplicity, low cost and is environmentally friendly. At the same time, less organic solvent was used in the proposed method. The proposed method accomplished low LODs, which are below the maximum residue limits (MRLs) of some neonicotinoid residues in agricultural products.

## Figures and Tables

**Table 1:** Analytical performance of the proposed method for five different neonicotinoids.

Insecticide	Linear Equation	Linearity (µg mL^−1^)	*r*^2^	Limit of Detection (LOD) (µg mL^−1^)	Limit of Quantification (LOQ) (µg mL^−1^)	% Relative Standard Deviation (RSD)		Enrichment Factor (EF)
						*t_R_*	Peak Area	
Thiamethoxam	*y* = 239217*x* + 1546.9	0.005–0.7	0.9978	0.001	0.003	1.02	3.77	100
Clothianidin	*y* = 834910*x* + 1822.5	0.005–0.7	0.9997	0.001	0.005	0.96	3.89	200
Imidacloprid	*y* = 505089*x* − 178.27	0.005–0.7	0.9999	0.0003	0.001	1.26	4.12	333
Acetamiprid	*y* = 805535*x* + 347.71	0.005–0.7	0.9999	0.0005	0.002	1.67	4.54	20
Thiacloprid	*y* = 2000006*x* + 5418.3	0.005–0.7	0.9992	0.002	0.006	1.92	0.01	100

**Table 2:** Recoveries of the studied neonicotinoids spiked in surface water samples obtained by the proposed method.

Analyte	Spiked (µg mL^−1^)	Surface Water I (*n* = 3)		Surface Water II (*n* = 3)		Surface Water III (*n* = 3)	
		Recovery (%)	RSD (%)	Recovery (%)	RSD (%)	Recovery (%)	RSD (%)
Thiamethoxam	0.00	ND	-	ND	-	ND	-
	0.05	101.89	2.45	89.50	1.21	98.12	0.95
	0.10	115.33	2.61	111.52	0.70	101.64	6.63
	0.25	116.72	2.14	115.52	1.84	117.56	4.20
Clothianidin	0.00	ND	-	ND	-	ND	-
	0.05	92.45	8.21	89.08	1.74	68.78	1.77
	0.10	112.22	1.57	100.31	1.55	91.50	9.63
	0.25	113.08	4.09	119.63	0.86	106.43	4.82
Imidacloprid	0.00	ND	-	ND	-	ND	-
	0.05	90.43	9.13	85.30	1.02	95.97	3.48
	0.10	106.23	1.34	95.04	1.18	90.64	9.35
	0.25	106.90	2.73	110.15	0.75	98.67	4.84
Acetamiprid	0.00	ND	-	ND	-	ND	-
	0.05	90.27	3.89	87.76	0.26	82.31	2.24
	0.10	110.31	0.64	100.07	1.06	92.19	9.08
	0.25	112.78	1.64	111.41	0.31	103.14	4.06
Thiacloprid	0.00	ND	-	ND	-	ND	-
	0.05	82.40	5.45	64.54	2.50	60.11	9.28
	0.10	88.64	0.55	88.64	3.99	88.64	13.71
	0.25	95.71	0.34	102.46	3.09	84.24	6.76

ND: not detected.

**Table 3:** Comparison of the proposed method with other sample preparation methods for the determination of neonicotinoids. Dispersive liquid-liquid microextraction (DLLME); quick, easy, cheap, effective, rugged and safe (QuEChERS); dispersive solid-phase extraction (DSPE); room-temperature ionic liquid-liquid-phase microextraction (RTIL-LPME); cloud point extraction (CPE); high performance liquid chromatography (HPLC); diode array (DAD); liquid chromatography (LC); mass spectrometry (MS); ultraviolet (UV).

Extraction Method	Analytical Technique	Linear Range	Recovery (%)	LOD	LOQ	EFs	Reference
DLLME	HPLC-DAD	LOQ–100.0 µg kg^−1^	73.4–118.3	1.5–2.5 µg kg^−1^	5.0–7.5 µg kg^−1^	66.6–105.9	[3]
QuEChERS	HPLC-DAD	LOQ–100.0 µg kg^−1^	73.8–89.9	2.0–2.5 µg kg^−1^	5.0–10.0 µg kg^−1^	72.2–85.2	[3]
DSPE (QuEChERS)	LC-MS/MS	0.005–0.5 µg mL^−1^	62.0–129.93	0.0007–0.002 µg mL^−1^	0.002–0.005 µg mL^−1^	-	[32]
DLLME	LC-MS/MS	LOQ–100.0 µg L^−1^	69.2–113.4	0.5–1.5 µg L^−1^	1.0–5.0 µg mL^−1^	67.8–95.0	[12]
QuEChERS	LC-MS/MS	LOQ–100.0 µg L^−1^	71.8–94.9	1.0–2.5 µg L^−1^	2.5–10.0 µg mL^−1^	51.3–96.2	[12]
DSPE and DLLME	HPLC-DAD	0.02–4.50 µg mL^−1^	76–123	0.002–0.005 µg mL^−1^	0.007–0.018 µg mL^−1^	-	[6]
RTIL-LPME	HPLC-DAD	0.41–5000 ng mL^−1^	69.2–113.4	0.12–0.33 ng mL^−1^	0.41–1.1 ng mL^−1^	655–843	[33]
Amended CPE	HPLC-UV	0.005–0.7 µg mL^−1^	60.12–117.57	0.0003–0.002 µg mL^−1^	0.001–0.006 µg mL^−1^	20–333	This work
